# Effectiveness of Growing-Up Milk Containing Only A2 β-Casein on Digestive Comfort in Toddlers: A Randomized Controlled Trial in China

**DOI:** 10.3390/nu15061313

**Published:** 2023-03-07

**Authors:** Ying Meng, Yubo Zhou, Hongtian Li, Yipu Chen, Grathwohl Dominik, Jie Dong, Youchi Tang, Jose M. Saavedra, Jianmeng Liu

**Affiliations:** 1 National Health Commission Key Laboratory of Reproductive Health/Institute of Reproductive and Child Health, Peking University Health Science Center, 38 Xueyuan Rd., Beijing 100191, China; 2 Department of Epidemiology and Biostatistics, School of Public Health, Peking University Health Science Center, 38 Xueyuan Rd., Beijing 100191, China; 3 Nestlé Product Technology Center—Nutrition, 1800 Vevey, Switzerland; 4 Nestlé Clinical Research Unit, 1000 Lausanne, Switzerland; 5 Wyeth Nutrition, Shanghai 200040, China; 6 Information Management Section, Chaoyang District Maternal and Child Health Hospital, Beijing 100021, China; 7 Division of Gastroenterology and Nutrition, Johns Hopkins University School of Medicine, Baltimore, MD 21287, USA

**Keywords:** A2 β-casein, digestive comfort, toddlers, randomized controlled trial, growing-up milk

## Abstract

Background: Emerging clinical evidence indicates the potential gastrointestinal (GI) benefits of milk containing only A2 β-casein, but data from randomized controlled trials is sparse among pediatric populations. We aimed to evaluate the effectiveness of growing-up milk (GUM) containing only A2 β-casein on GI tolerance in toddlers. Methods: A total of 387 toddlers aged 12–36 months were recruited in Beijing, China, and randomized in a 1:1:1 ratio to consume one of two commercially available A2 GUMs (combined in the analysis as A2 GUM) or continue their current feeding regimen of conventional milk for 14 days. The primary outcome was the total Gut Comfort Score (GCS) (range: 10–60; higher values indicate greater GI distress) derived from a 10-item (score range: 1–6 per item) parent-reported questionnaire, reflecting GI tolerance. Results: The GCS (mean ± SD) was comparable between the A2 GUM and conventional milk groups on day 7 (14.7 ± 5.0 vs. 15.0 ± 6.1, *p* = 0.54) and day 14 (14.0 ± 4.5 vs. 14.3 ± 5.5, *p* = 0.51). Parents reported less constipation in those consuming A2 GUM vs. conventional milk on day 14 (1.3 ± 0.6 vs. 1.4 ± 0.9, *p* = 0.020). Among 124 participants with minor GI distress at baseline (GCS ≥ 17, top tertile range 17–35), GCS was significantly lower in those consuming A2 GUM on day 7 (18.2 ± 5.1 vs. 21.2 ± 6.8, *p* = 0.004) and day 14 (17.1 ± 5.3 vs. 19.6 ± 6.3, *p* = 0.026), as were individual GI symptoms (all *p* < 0.05). In the toddlers without GI issues at baseline (GCS < 17), a low GCS was maintained throughout the study period after switching to A2 GUM (mean values range 10–13). Conclusions: Growing-up milk containing only A2 β-casein were well-tolerated and associated with lower parent-reported constipation scores after two weeks when compared to conventional milks. In healthy toddlers with minor GI distress, A2 GUM improved overall digestive comfort and GI-related symptoms within one week.

## 1. Introduction

Functional gastrointestinal disorders (FGIDs), a mixture of age-dependent, chronic or recurrent symptoms without evident structural or biochemical abnormalities [1], are common in infants and young children. The prevalence of FGIDs in infants was reported 30% worldwide in 2016 [2], 27% in the US in 2015 [3], and 34% in China in 2014 [4]. The symptoms of FGIDs, such as colic and functional diarrhea, were clearly defined in Rome IV criteria [5]. When the symptoms are mild and do not reach the strict diagnostic criteria for FGID, they are sometimes labeled as mild gastrointestinal disorders (MGDs). MGDs are very common among pediatric populations [6,7] and are associated with not only discomfort in children but also distress to their parents. However, MGDs often go untreated since they do not fulfill any clinical diagnostic criteria. Therefore, some innovations in pediatric nutritional solutions have been focused on alleviating GI distress in children suffering from MGDs.

Milk is an important part of toddler diets and is made up of whey and casein fractions. β-casein, comprising up to 45% of total caseins, is a phosphorylated protein, and could be digested into bioactive peptides, causing digestive and metabolic, hormone, immune, neural, and behavioral responses [8,9]. Cow’s milk generally contains two types of β-casein, A1 and A2 β-casein, whereas human milk contains only A2 β-casein [10]. A1 β-casein is hydrolyzed to β-casomorphin-7 (BCM-7), which affects the endocrine and immune systems by activating GI opioid receptors, resulting in decreased GI motility and increased GI transit time [11,12]. Therefore, it has been suggested that A1 β-casein may be associated with GI intolerance. In contrast, A2 β-casein does not generate BCM-7, suggesting that milk containing only A2 β-casein may be associated with better GI motility and reduced GI symptoms compared with milk containing both A1 and A2 β-caseins [11,12].

Several studies from developed and developing countries have shown GI improvement of A2 milk intake among adults [13,14,15,16]. However, only two studies with limited sample sizes (*n* ≤ 80) have assessed the health effects of A2 versus A1 β-casein among children and reported inconsistent results. A double-blind crossover study of children aged 21 months to 12 years with chronic functional constipation showed no difference in the resolution of constipation between those consuming A1 and A2 β-casein milk [17]. However, a recent randomized, crossover study of children aged 5–6 years old with milk intolerance showed that consumption of A2 β-casein milk versus conventional milk was associated with reduced parent-reported GI symptoms [18]. We are not aware of any studies that have evaluated the effects of A2 β-casein milk in healthy toddlers.

The objective of this study was to compare the signs and symptoms of GI tolerance between toddlers aged 12–36 months consuming growing-up milk (GUM) containing only A2 β-casein and those consuming conventional milk containing both A1 and A2 β-casein.

## 2. Materials and Methods

### 2.1. Study Setting and Design

This randomized, controlled, open-label study was conducted in a community healthcare center in Beijing, China, from September 2018 to January 2019. Healthy toddlers aged 12–36 months were recruited by trained physicians and randomized to receive one of two A2 GUM brands that were commercially available in China at the time of the study start (A2 GUM group A and A2 GUM group B) or to the conventional milk group for 14 consecutive days. The nutritional compositions of both A2 GUMs were comparable and similar; therefore, the two A2 GUM groups were combined as the A2 GUM group in the analysis below. This study was approved by the Peking University Institutional Review Board (IRB00001052-18063). All participants’ parents or caregivers completed an informed consent form.

### 2.2. Participants

To be eligible for the study, toddlers (1) were born full-term (37–42 weeks gestation), (2) had a birth weight of 2.5 to 4.5 kg, and (3) were habitually consuming any cow’s milk, conventional milks, and/or dairy products and (4) were 12–36 months of age. Exclusion criteria were (1) chronic infectious, metabolic, genetic, or other diseases potentially impacting GI function or feeding practices, (2) known cognitive and developmental disorders, or (3) currently using or had ever used therapeutic infant formulas (i.e., hypoallergenic, lactose-free, or anti-regurgitation formulas).

### 2.3. Randomization and Interventions

At enrollment, the physician received an instant WeChat Quick Response (QR) code from the randomization schedule, which was scanned by participants to receive their allocation information. A random block size of 9 was applied to guarantee balanced allocation. Allocation was masked from the participants and physicians until baseline information was completely collected. All participant data and allocation information were stored on a secure server hosted by the Peking University Maternal and Child Health WeChat platform, compliant with local data security requirements. After enrollment, participants were randomized to A2 GUM group A, A2 GUM group B, or the conventional milk group in a 1:1:1 ratio. Participants in A2 GUM group A received formula A (Askeaton, Ireland), and those in A2 GUM group B received formula B (Shanghai, China) in an open-label manner. Both A2 formulas are GUMs designed for toddlers aged 12 months or older, and all β-casein proteins the formulas contained are of A2 origin. Based on the nutritional information provided on the product label, the nutritional composition of both A2 formulas, including total protein and lactose levels and the presence of prebiotics, are comparable. Toddlers in the A2 GUM groups were instructed to consume at least 300 mL of the study milks per day (two servings, 150 mL each) during the 14-day intervention period, and those in the conventional milk group maintained their habitual diet. Milk intake was assessed using a parent-reported milk intake diary at baseline, and on days 5–7 and 12–14 to record the type and volume of milk consumed over a 24 h period.

### 2.4. Study Outcomes

The primary outcome was the total GCS, evaluated using a parent-reported toddler gut comfort questionnaire. The questionnaire was administered at baseline, on three consecutive days from days 5–7, and on three consecutive days from days 12–14, to collect the prevalence and severity of gastrointestinal symptoms of toddlers. At baseline, parents completed the questionnaires under the guidance of physicians who had been unanimously trained on the content the questionnaire to minimize potential bias. Afterwards, parents completed the questionnaires by themselves. The questionnaire was modeled after a previously reported standardized infant gastrointestinal symptom questionnaire (IGSQ-13). It included ten questions related to GI symptoms (6 questions for stooling issues, constipation, diarrhea, gassiness, abdominal pain, and bloated) and related behaviors (4 questions for irritability, sleep problems, sleep duration during the day, and times of waking up during the night). The score for each individual question ranged from 1 to 6, assigned using a visual analog scale, leading to a total GCS ranging from 10 to 60. A higher score indicated a greater GI distress.

Secondary outcomes included stool characteristics, temperament, anthropometric parameters, and adverse events. Stool frequency and consistency were reported by parents prospectively over a 24 h period using a toddler stool diary at baseline, days 5–7, and days 12–14. Stool consistency was assessed using a validated 5-point scale (1 = watery, 2 = runny, 3 = mushy soft, 4 = formed, or 5 = hard) [19]. Temperament over the intervention period was assessed using a simplified 9-item Early Childhood Behavior Questionnaire [20] at day 14, which included attention focusing (1 question), activity level (1 question), sociability (2 questions), effortful control (1 question), irritability (2 questions), and soothability (2 questions). The score of each question ranged from 1 to 7 according to the frequency of behaviors observed by parents, with higher scores indicating higher frequency. Anthropometric parameters, including weight, length, and head circumference, were measured at clinic visits by experienced physicians at baseline and day 14 using standard methods. BMI (kg/m^2^) was calculated as weight in kilograms divided by squared height in meters. The Z-score of each parameter was calculated according to the WHO child growth standards according to age and sex [21]. Adverse events (AEs), including incidence, severity, seriousness, and relation to study formula consumption, as well as concomitant medications and treatments, were reported by parents and checked by physicians throughout the study period. Predetermined GI AEs of interest included hard stool, constipation, difficulty with bowel movement, acute diarrhea, chronic diarrhea, and gastroesophageal reflux disease.

### 2.5. Sample Size

According to previous data collected using the Infant Gastrointestinal Symptom Questionnaire (IGSQ-13) [22,23,24], with a 2-sided significance level of *p* = 0.05 and 80% power, assuming a dropout rate of 10%, approximately 360 toddlers in total were needed to detect a mean difference of 1.25 points in the GCS (10% reduction from baseline mean GCS, and a standard deviation of 4.2) between the A2 GUM and conventional milk groups.

### 2.6. Statistical Analyses

Continuous variables were represented using the mean (standard deviation, SD) or median (interquartile ranges, IQR), and categorical variables were represented using frequency (percentage). Differences between milk groups were explored by *t*-test for means, Kruskal–Wallis test for medians, and chi-square test for frequencies. The average GCS and stool frequency and consistency scores at days 5–7 and days 12–14 were calculated to reflect digestive comfort or stool characteristics at day 7 and day 14, also for the following analyses. Multivariate-adjusted mean differences (95% CI) were calculated using analysis of covariance, adjusting for the baseline total GCS, intervention group, age at enrollment, sex, and whether or not they were ever breastfed. If a significant modification effect by baseline GCS was observed, subgroup analyses were then conducted within strata of baseline scores by tertiles, with participants in the top tertile defined as having minor GI distress. Independent t-tests were used to compare stool frequency and consistency, temperament, and anthropometric parameters (weight, length, head circumference, BMI, and Z-scores) between the two groups. A chi-square test was used to compare adverse events between groups. All analyses were based on the intention-to-treat principle using the full analysis set (FAS). Sensitivity analyses of the primary outcome were further conducted in the per-protocol set (PPS). All *p* values were 2-sided, and statistical significance was set at *p* < 0.05. All analyses were conducted using SAS/STAT software (version 9.4, SAS Institute, Inc., Cary, NC, USA).

## 3. Results

### 3.1. Baseline Characteristics and Milk Intake

**Figure 1:**
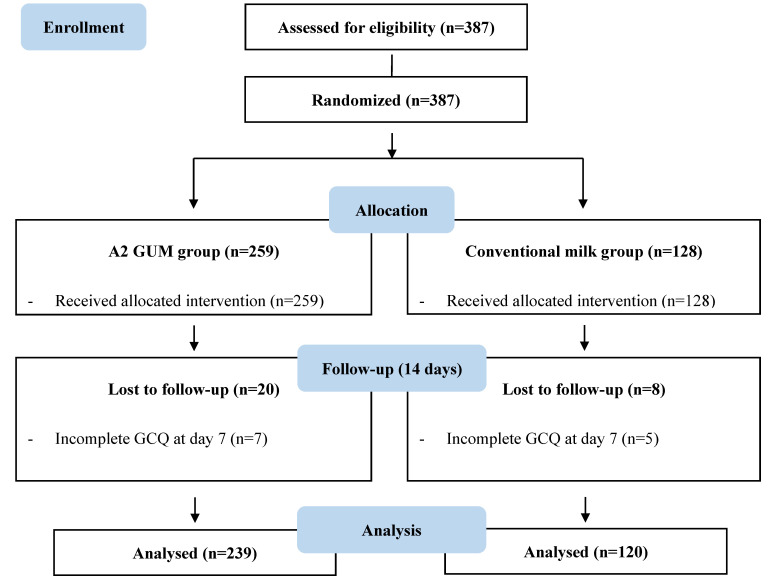
Flow chart.

Of 387 enrolled toddlers, 359 completed the study. A total of 259 participants were allocated to the A2 GUM group, and 128 were allocated to the conventional milk group (Figure 1). All baseline demographic characteristics of the participants were comparable between the two groups (Table 1).

On day 7 and day 14, the daily A2 GUM intake (mean ± SD) was 285 ± 144 mL and 285 ± 142 mL, respectively. The details of milk intake between the A2 GUM and conventional milk groups are presented in Table A1.

### 3.2. Digestive Comfort

The total GCS (mean ± SD) was 15.2 ± 5.5 at baseline, 14.8 ± 5.4 on day 7, and 14.1 ± 4.9 on day 14, indicating the maintenance of good digestive comfort in these healthy toddlers. There were no significant differences in total GCS between the A2 GUM and conventional milk groups at baseline (15.1 ± 5.5 vs. 15.2 ± 5.5, *p* = 0.91), on day 7 (14.7 ± 5.0 vs. 15.0 ± 6.1, *p* = 0.54), or on day 14 (14.0 ± 4.5 vs. 14.3 ± 5.5, *p* = 0.51). Parents reported less constipation in the A2 GUM group than in the conventional milk group on day 14 (1.3 ± 0.6 vs. 1.4 ± 0.9, *p* = 0.020). There were no significant differences in other GI symptoms or related behaviors between groups, except for the number of waking-ups during the night (2.2 ± 1.0 in the A2 GUM group vs. 1.9 ± 0.9 in the conventional milk group, *p* = 0.009). The interaction term between feeding group and baseline GCS was statistically significant (*p* = 0.035) in the multivariate linear regression model with GCS on day 14 as the outcome, providing justification for stratifying the analysis by tertiles of baseline GCS score. The cutoff score for the top tertile was 17 points, and participants with total GCS ≥ 17 at baseline (range 17–35) were defined as having minor GI distress. The bottom two tertiles were grouped together in the analysis due to the homogenous spread of scores (range 10–16).

**Figure 2:**
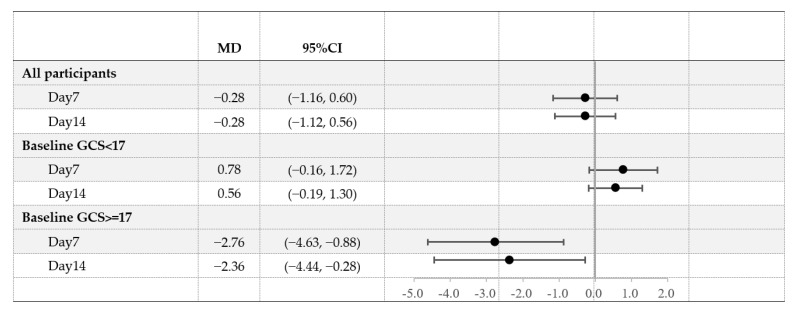
Least-square means (LS means) and 95% confidence intervals (CI) for Gut Comfort Composite Score (GCS) by baseline GCS tertiles and group, day 7 and day 14. Models included adjustment for baseline GCS, age, sex, and breastfeeding. A negative value indicates that the GCS of the A2 GUM group was lower than that of the conventional milk group.

Among participants with total GCS < 17 at baseline, no significant difference was observed in total GCS or individual scores between the two groups, and the scores were consistently low over the study period. Among toddlers with minor GI distress (total GCS ≥ 17 at baseline), the scores at baseline were comparable between groups. However, on day 7, those consuming A2 GUM versus those consuming conventional milk had significantly improved overall gastrointestinal symptoms (18.2 ± 5.1 vs. 21.2 ± 6.8, *p* = 0.004), constipation (1.8 ± 0.9 vs. 2.3 ± 1.1, *p* = 0.001), diarrhea (1.6 ± 0.6 vs. 1.9 ± 0.7, *p* = 0.004), gassiness (1.7 ± 0.7 vs. 2.1 ± 0.8, *p* = 0.002), abdominal pain (1.5 ± 0.6 vs. 1.8 ± 0.7, *p* = 0.003), bloating (1.6 ± 0.6 vs. 2.0 ± 0.8, *p* = 0.002), and fussiness and irritability (1.7 ± 0.7 vs. 2.3 ± 1.1, *p* = 0.014) (Table 2). The same pattern was observed on day 14. The mean difference (MD) between the A2 GUM and conventional milk groups in the change in GCS on days 7 and 14 from baseline was significantly greater for participants in the top tertile than for participants in the bottom tertiles (MD −2.76 vs. 0.78 points on day 7 and −2.36 vs. 0.56 points on day 14, Figure 2). Analyses limited to the PPS population showed similar results to the FAS population (Table A2).

### 3.3. Stool Characteristics

Stool consistency was comparable between the A2 GUM group and the conventional milk group at baseline (3.9 ± 0.6 vs. 3.8 ± 0.6, *p* = 0.27), on day 7 (3.9 ± 0.6 vs. 3.8 ± 0.6, *p* = 0.64), and day 14 (3.9 ± 0.6 vs. 3.8 ± 0.6, *p* = 0.62). Stool frequency was higher in the A2 GUM group at baseline (2.5 ± 1.2 vs. 1.3 ± 0.6, *p* < 0.001) and remained higher on days 7 and 14 (2.9 ± 1.2 vs. 1.6 ± 0.8, *p* < 0.001 and 2.7 ± 1.2 vs. 1.3 ± 0.6, *p* < 0.001, respectively).

### 3.4. Temperament Characteristics

Toddlers in the A2 GUM group had better scores than those in the conventional milk group for sociability (4.9 ± 1.7 vs. 4.7 ± 1.8, *p* = 0.028; 4.5 ± 1.8 vs. 4.2 ± 1.9, *p* = 0.047) and soothability (4.5 ± 1.6 vs. 4.2 ± 1.6, *p* = 0.004) but were less active (3.1 ± 1.7 vs. 3.4 ± 1.8, *p* = 0.030) (Table 3). No significant difference was observed for other temperament items.

### 3.5. Anthropometric Parameters

There were no significant differences in any anthropometric parameters on day 14 between the two groups (Table 4).

### 3.6. Adverse Events

The overall incidence of AEs was low and comparable between the groups (Table 5). In the A2 GUM group, a total of 19 events occurred in 10 (3.9%) participants, while a total of 8 events occurred in 5 (3.9%) participants in the conventional milk group. Of the predetermined GI AEs of interest, only constipation and diarrhea were observed, both with very low incidence and with no significant difference between the groups.

## 4. Discussion

In this randomized controlled study of healthy toddlers aged 12 to 36 months old, we found that cow’s milk-based formulas containing only A2 β-casein were easy-to-digest and reduced parent-reported constipation compared to conventional formulas during the 14-day intervention period. Among toddlers with minor gastrointestinal distress, A2 GUM improved overall digestive comfort and gastrointestinal related symptoms, within one week of intervention.

In our population, low gut comfort scores at baseline indicated that the overall digestive tolerance was overall good at enrollment and was well maintained over the entire intervention period. We did not observe significant differences in overall digestive comfort or in most individual GI symptoms between the toddlers in the A2 GUM group and those in the conventional milk group, possibly due to the good digestive comfort of the study population at baseline and the relatively short intervention period. These findings suggested that the A2 GUM was as well tolerated as conventional milk in healthy toddlers with good digestive health. We found that parents tended to report less constipation of their toddlers in the A2 GUM group versus those in the conventional milk group on day 14. Constipation is most common during toddlerhood, with the prevalence as high as 30% [25,26,27]. Children with constipation reported greater impairment in quality of life than children with other gastrointestinal complaints [28,29], and might even have behavioral, social, and emotional problems [30]. A recent cross-over study among Chinese children aged 5–6 years with lactose intolerance demonstrated that those consuming A2 β-casein for five days were more likely to have significantly softer stools [19], similar to our findings. Another study conducted among Australian children aged 21–144 months showed a higher resolution of the proportion of constipation in A2 versus in A1 milk group (79% vs. 57%), but the proportions did not differ statistically possibly due to the limited sample size (*n* = 39) [17]. Hence, it is likely that the consumption of A2 GUM benefits toddlers with constipation. In addition, the number of waking-ups during the night was higher in A2 GUM group than conventional milk group, which was possibly due to the adaption of a new milk for toddlers in A2 GUM group [31].

It is worth noting that in this study, among toddlers in the top tertile of GCS who experienced minor gastrointestinal distress, there were greater improvements in overall digestive comfort and individual symptoms including constipation, diarrhea, gassiness, and abdominal pain, as soon as after 7 and 14 days of consumption. These findings were in line with previous studies which showed significant effects among participants with greater gastrointestinal distress [16,17,32]. Randomized controlled trials conducted among lactose-intolerant adults or children from the United States, Australia, and China, refs. [13,14,15,33], showed that milk containing only A2 β-casein improved GI symptoms and reduced digestive discomfort.

The digestive comfort of children might influence their temperament, including mood, behavior problems, and temper tantrums [34,35]. Previous findings also showed that physical discomfort was associated with negative emotionality and temperament [20,36,37]. Our data on temperament showed that toddlers consuming A2 GUM were more sociable and more easily soothed but less active than those consuming conventional milk.

One of the potential mechanisms underlying the effect of formulas containing only A2 versus those containing a mixture of A1 and A2 β-casein on digestive comfort is that the digestion of A1, but not A2, β-casein can produce BCM-7. In vivo studies in both humans and animals have shown that BCM-7 decreases GI motility and increases GI transit time [11,14,38], thus contributing to hard and dry stools, leading to GI symptoms such as constipation and abdominal pain. Additionally, BCM-7 was associated with changes in inflammatory responses and increased serum or luminal myeloperoxidase activity and concentrations of IL-4 and histamine, which can contribute to GI inflammation [11]. Previous studies have also demonstrated lower concentrations of BCM-7 or inflammatory biomarkers in adults who consumed milk containing only A2 β-casein compared to those who consumed milk containing both A1 and A2 β-casein [13,14,16].

Our findings among healthy toddlers may have public health and clinical relevance given the high prevalence of GUM consumption in pediatric populations. The prevalence is 75% in Chinese toddlers aged 12–23 months, and remains to be over 50% up to 35 months of age [39]. The strengths of this study include the prospective and randomized approach with a clear documentation of the amounts of milk consumed by each participant. Parent reports based on validated and published questionnaires on digestive comfort and temperament covering a variety of domains were utilized, and the relatively large sample size enabled us to perform subgroup analysis. Nevertheless, our study also had some limitations. First, we employed a relatively short feeding and follow-up period (2 weeks), which limited the examination of the potential long-term effects of A2 milk consumption. However, the GI benefits manifested after a short period of consumption of A2 GUM particularly in toddlers with minor GI distress at baseline in this study, within just one week. Second, the average A2 GUM intakes on day 7 and day 14 were slightly lower than recommended (285 mL vs. 300 mL), which may underestimate the effectiveness of A2 GUM. Third, data on complementary feeding other than milk products were not collected. Nevertheless, the RCT design was adopted to minimize potential bias introduced by complementary foods. Lastly, although participants were randomized to the study groups, the intervention was not blinded to them.

## 5. Conclusions

In conclusion, we found that cow’s milk-based formulas containing only A2 β-casein were well-tolerated overall and reduced parent-reported constipation scores compared to conventional milks after two weeks. In healthy toddlers with minor GI distress at baseline, A2 GUM improved overall digestive comfort and GI-related symptoms, and improvements were seen within one week of the feeding intervention. The findings indicate that such formulas containing only A2 β-casein were as well tolerated as conventional milks for healthy toddlers in GI tolerance, but likely benefit to improve constipation or minor GI distress. Further studies with longer follow-up periods in diverse populations are warranted to confirm these findings and to investigate potential long-term beneficial effects of consuming milk products containing only A2 β-casein.

## Figures and Tables

**Table 1:** Demographic and anthropometric characteristics of toddlers at baseline.

**Characteristics**	**Total** **(*n* = 387)**	**A2 GUM** **(*n* = 259)**	**Conventional Milk** **(*n* = 128)**	***p*-Value**
Age (months)	23.6 ± 6.6	23.3 ± 6.5	24.1 ± 6.8	0.225
Sex, *n* (%)				0.637
Male	199 (51.4)	131 (50.6)	68 (53.1)	
Female	188 (48.6)	128 (49.4)	60 (46.9)	
Parity, *n* (%)				0.750
Primipara	268 (69.3)	178 (68.7)	90 (70.3)	
Multipara	119 (30.7)	80 (31.3)	38 (29.7)	
Delivery mode, *n* (%)				0.157
Vaginal delivery	237 (61.2)	165 (63.7)	72 (56.2)	
Cesarean delivery	150 (38.8)	94 (36.3)	56 (43.8)	
Family history of digestive diseases	1 (0.3)	0 (0)	1 (0.8)	0.331
Breastfeeding				0.150
Still breastfeeding	78 (20.2)	50 (19.3)	28 (21.9)	
Ever breastfed	292 (75.5)	194 (74.9)	98 (76.6)	
Never breastfed	17 (4.4)	15 (5.8)	2 (1.6)	
Breastfeeding duration, months	14.2 ± 6.5	13.9 ± 6.2	14.7 ± 6.6	0.260

**Table 2:** GI symptom scores from the Toddler Gut Comfort Questionnaire at baseline, day 7, and day 14 among all participants and stratified by baseline Gut Comfort Score (GCS).

Toddler Gut Comfort Questionnaire Item (Mean ± SD)	All Participants				Baseline GCS < 17			Baseline GCS >= 17		
	Total	A2 GUM	Conventional Milk	*p*	A2 GUM	Conventional Milk	*p*	A2 GUM	Conventional Milk	*p*
	(*n* = 387)	(*n* = 259)	(*n* = 128)		(*n* = 175)	(*n* = 88)		(*n* = 84)	(*n* = 40)	
**BASELINE**										
**Total GCS**	15.2 ± 5.5	15.1 ± 5.5	15.2 ± 5.5	0.91	12.0 ± 1.7	12.0 ± 1.8	0.92	21.7 ± 4.9	22.2 ± 4.4	0.60
**Gastrointestinal Symptoms**										
Stooling issues *	1.5 ± 0.8	1.5 ± 0.8	1.5 ± 0.9	0.75	1.1 ± 0.3	1.1 ± 0.4	0.17	2.3 ± 1.0	2.3 ± 1.0	0.99
Constipation *	1.5 ± 1.0	1.6 ± 1.0	1.5 ± 0.9	0.41	1.1 ± 0.4	1.1 ± 0.2	**0.042**	2.5 ± 1.3	2.4 ± 1.2	0.91
Diarrhea *	1.3 ± 0.6	1.3 ± 0.6	1.3 ± 0.6	0.95	1.1 ± 0.2	1.1 ± 0.3	0.43	1.9 ± 0.7	1.9 ± 0.8	0.90
Gassiness *	1.4 ± 0.7	1.3 ± 0.6	1.4 ± 0.8	0.42	1.0 ± 0.2	1.1 ± 0.3	0.14	2.0 ± 0.9	2.2 ± 0.9	0.42
Abdominal pain *	1.3 ± 0.6	1.3 ± 0.6	1.2 ± 0.5	0.74	1.0 ± 0.2	1.1 ± 0.3	0.20	1.8 ± 0.8	1.7 ± 0.6	0.45
Bloating †	1.4 ± 0.7	1.4 ± 0.8	1.4 ± 0.7	0.84	1.0 ± 0.1	1.0 ± 0.2	0.21	2.1 ± 1.0	2.1 ± 0.9	0.82
**Gastrointestinal-related Behaviors**										
Fussy and irritable *	1.6 ± 0.9	1.5 ± 0.8	1.6 ± 1.0	0.29	1.2 ± 0.6	1.2 ± 0.4	0.40	2.1 ± 0.9	2.6 ± 1.2	**0.016**
Sleep problems ‡	1.5 ± 0.8	1.5 ± 0.8	1.6 ± 0.9	0.64	1.2 ± 0.4	1.2 ± 0.5	0.91	2.2 ± 0.9	2.4 ± 1.0	0.32
Sleepy during the day *	1.6 ± 0.8	1.6 ± 0.8	1.5 ± 0.7	0.95	1.2 ± 0.5	1.2 ± 0.5	0.81	2.2 ± 0.8	2.2 ± 0.8	0.93
Waking up during the night §	2.2 ± 1.0	2.2 ± 1.1	2.1 ± 0.9	0.51	2.0 ± 0.9	2.0 ± 0.9	0.81	2.6 ± 1.2	2.5 ± 1.0	0.50
**DAY 7**										
**Total GCS**	14.8 ± 5.4	14.7 ± 5.0	15.0 ± 6.1	0.54	13.0 ± 4.0	12.2 ± 2.7	0.11	18.2 ± 5.1	21.2 ± 6.8	**0.004**
**Gastrointestinal Symptoms**										
Stooling issues *	1.5 ± 0.9	1.5 ± 0.9	1.5 ± 0.8	0.70	1.3 ± 0.7	1.1 ± 0.3	0.09	1.9 ± 1.0	2.2 ± 1.1	0.076
Constipation *	1.5 ± 0.8	1.5 ± 0.8	1.5 ± 0.9	0.32	1.3 ± 0.6	1.1 ± 0.3	0.07	1.8 ± 0.9	2.3 ± 1.1	**0.001**
Diarrhea *	1.3 ± 0.5	1.3 ± 0.5	1.4 ± 0.6	0.057	1.1 ± 0.4	1.1 ± 0.3	0.90	1.6 ± 0.6	1.9 ± 0.7	**0.004**
Gassiness *	1.4 ± 0.7	1.3 ± 0.6	1.5 ± 0.9	0.19	1.1 ± 0.4	1.1 ± 0.3	0.34	1.7 ± 0.7	2.1 ± 0.8	**0.002**
Abdominal pain *	1.3 ± 0.5	1.3 ± 0.5	1.3 ± 0.6	0.22	1.1 ± 0.4	1.1 ± 0.3	0.28	1.5 ± 0.6	1.8 ± 0.7	**0.003**
Bloating †	1.3 ± 0.6	1.3 ± 0.5	1.4 ± 0.7	0.10	1.1 ± 0.4	1.1 ± 0.3	0.67	1.6 ± 0.6	2.0 ± 0.8	**0.002**
**Gastrointestinal-related Behaviors**										
Fussy and irritable *	1.4 ± 0.7	1.4 ± 0.7	1.5 ± 0.8	0.40	1.3 ± 0.6	1.2 ± 0.4	0.40	1.7 ± 0.7	2.2 ± 1.1	**0.014**
Sleep problems ‡	1.4 ± 0.7	1.4 ± 0.7	1.5 ± 0.7	0.38	1.2 ± 0.5	1.2 ± 0.3	0.53	1.9 ± 0.9	2.2 ± 0.8	**0.033**
Sleepy during the day *	1.5 ± 0.7	1.5 ± 0.7	1.5 ± 0.6	0.83	1.3 ± 0.5	1.2 ± 0.4	0.42	2.0 ± 0.7	2.1 ± 0.7	0.17
Waking up during the night §	2.2 ± 1.0	2.3 ± 1.0	2.1 ± 0.9	**0.026**	2.1 ± 0.9	1.9 ± 0.8	0.053	2.6 ± 1.2	2.3 ± 1.0	0.29
**DAY 14**										
**Total GCS**	14.1 ± 4.9	14.0 ± 4.5	14.3 ± 5.5	0.51	12.5 ± 3.1	11.9 ± 2.7	0.15	17.1 ± 5.3	19.6 ± 6.3	**0.026**
**Gastrointestinal Symptoms**										
Stooling issues *	1.4 ± 0.8	1.4 ± 0.7	1.5 ± 0.9	0.29	1.2 ± 0.4	1.1 ± 0.5	0.53	1.8 ± 1.0	2.1 ± 1.1	**0.030**
Constipation *	1.3 ± 0.7	1.3 ± 0.6	1.4 ± 0.9	**0.020**	1.2 ± 0.5	1.2 ± 0.5	0.74	1.7 ± 0.8	2.1 ± 1.2	**0.008**
Diarrhea *	1.2 ± 0.5	1.2 ± 0.5	1.3 ± 0.5	0.31	1.1 ± 0.3	1.1 ± 0.3	0.28	1.5 ± 0.6	1.8 ± 0.7	**0.014**
Gassiness *	1.2 ± 0.5	1.2 ± 0.5	1.3 ± 0.6	0.16	1.1 ± 0.3	1.1 ± 0.2	0.47	1.5 ± 0.7	1.9 ± 0.8	**0.008**
Abdominal pain *	1.2 ± 0.4	1.2 ± 0.4	1.3 ± 0.5	0.19	1.1 ± 0.3	1.1 ± 0.2	0.29	1.4 ± 0.6	1.2 ± 0.5	**0.018**
Bloating †	1.3 ± 0.5	1.3 ± 0.5	1.3 ± 0.6	0.070	1.1 ± 0.3	1.1 ± 0.3	0.74	1.6 ± 0.7	1.9 ± 0.8	**0.005**
**Gastrointestinal-related Behaviors**										
Fussy and irritable *	1.4 ± 0.7	1.4 ± 0.7	1.4 ± 0.7	0.89	1.2 ± 0.6	1.1 ± 0.3	0.34	1.7 ± 0.8	2.0 ± 0.8	0.24
Sleep problems ‡	1.4 ± 0.7	1.4 ± 0.7	1.4 ± 0.6	0.99	1.2 ± 0.5	1.2 ± 0.3	0.32	1.8 ± 0.8	2.0 ± 0.8	0.25
Sleepy during the day *	1.4 ± 0.6	1.4 ± 0.6	1.4 ± 0.6	0.66	1.2 ± 0.4	1.2 ± 0.3	0.42	1.8 ± 0.7	1.9 ± 0.6	0.16
Waking up during the night §	2.2 ± 1.0	2.2 ± 1.0	1.9 ± 0.9	**0.009**	2.1 ± 1.0	1.9 ± 0.9	**0.030**	2.4 ± 1.1	2.1 ± 0.8	0.16

* 1-Never; 6-Always; † 1-None; 6-Very strong; ‡ 1-Not a problem at all; 6-A very serious problem; § 1-Never; 2-Once; 3-Twice; 4-Three Times; 5-Four times; 6-More than four times. *p* values < 0.05 were marked in bold.

**Table 3:** Temperament items at day 14, by group.

Temperament Question	A2 GUM	Conventional Milk	*p*
**Irritability**: When having trouble completing a task (e.g., building, drawing, dressing), how often did your child get easily irritated?	5.2 ± 1.4	5.4 ± 1.6	0.66
**Irritability**: When s/he asked for something and you said “no”, how often did your child have a temper tantrum?	4.2 ± 1.6	4.5 ± 1.6	0.37
**Attention focusing**: When engaged in play with his/her favorite toy, how often did your child play for more than 10 min?	4.8 ± 1.8	4.5 ± 1.9	0.061
**Effortful control**: During everyday activities, how often did your child pay attention to you right away when you called to him or her?	4.8 ± 1.7	4.5 ± 1.9	0.099
**Sociability**: When a familiar adult, such as a relative or friend, visited your home, how often did your child want to interact with the adult?	4.9 ± 1.7	4.7 ± 1.8	**0.028**
**Sociability**: When a familiar child came to your home, how often did your child seek out the company of the child?	4.5 ± 1.8	4.2 ± 1.9	**0.047**
**Activity level**: While playing indoors, how often did your child run through the house?	3.1 ± 1.7	3.4 ± 1.8	**0.030**
**Soothability**: When s/he was upset, how often did your child cry for more than 3 min, even when being comforted?	5.1 ± 1.6	5.2 ± 1.4	0.99
**Soothability**: When s/he was upset, how often did your child become easily soothed?	4.5 ± 1.6	4.2 ± 1.6	**0.004**

1-Never; 2-Very rarely; 3-Less than half the time; 4-About half the time; 5-More than half the time; 6-Almost always; 7-Always. *p* values < 0.05 were marked in bold.

**Table 4:** Anthropometric parameters at baseline and day 14.

	A2 GUM	Conventional Milk	*p*
**BASELINE**			
Weight (kg)	12.61 ± 2.36	12.44 ± 2.11	0.50
Weight (Z-score)	0.63 ± 1.25	0.41 ± 0.94	0.08
Length (cm)	86.11 ± 7.03	87.11 ± 7.09	0.19
Length (Z-score)	0.20 ± 1.35	0.30 ± 1.31	0.52
Head circumference (cm)	47.54 ± 3.17	47.80 ± 2.83	0.44
Head circumference (Z-score)	0.06 ± 2.20	0.15 ± 1.78	0.70
BMI (kg/m^2^)	17.03 ± 2.86	16.39 ± 1.88	0.02
BMI (Z-score)	0.73 ± 1.75	0.32 ± 1.26	0.02
**DAY 14**			
Weight (kg)	13.09 ± 2.85	13.11 ± 2.59	0.95
Weight (Z-score)	0.85 ± 1.36	0.77 ± 1.34	0.67
Length (cm)	86.78 ± 6.97	87.27 ± 7.11	0.56
Length (Z-score)	0.29 ± 1.23	0.24 ± 1.32	0.75
Head circumference (cm)	48.27 ± 2.36	47.93 ± 3.06	0.27
Head circumference (Z-score)	0.54 ± 1.61	0.21 ± 2.08	0.13
BMI (kg/m^2^)	17.34 ± 2.91	17.21 ± 2.81	0.72
BMI (Z-score)	0.94 ± 1.79	0.87 ± 1.71	0.75

**Table 5:** Adverse events during the study period.

	A2 GUM			Conventional		Milk	*p*
	Participants (*n*)	% Events (*n*)		Participants (*n*)	%	Events (*n*)	
All Adverse Events	10	3.9	19	5	3.9	8	>0.999
Individual Adverse Events							
Weepiness or crying for no apparent reason	2	0.8	2	0	0	0	>0.999
Fever	2	0.8	2	1	0.8	1	0.549
Constipation	1	0.4	1	0	0	0	>0.999
Hard stool	1	0.4	3	1	0.8	1	0.553
Diarrhea	1	0.4	1	0	0	0	>0.999
Respiratory infection	3	1.2	3	3	2.3	3	0.875
Others	4	1.5	7	2	1.6	3	0.705

## Data Availability

Data described in the manuscript, code book, and analytic code will not be made available because specific consent has not been obtained from neither participants nor ethics committee approving the study at the time of study conduct.

## Appendix A

**Table A1:** Milk intake in the past 24 h among FAS participants.

	A2 GUM			Conventional Milk		
	Baseline	Day 7	Day 14	Baseline	Day 7	Day 14
A2 GUM/mL	75 ± 152	285 ± 144	285 ± 142	/	/	/
GUM/mL	112 ± 173	40 ± 110	36 ± 108	119 ± 175	269 ± 165	276 ± 167
Cow’s milk/mL	11	8	8	7	26	21
Fortified cow’s milk/mL	2	0	2	1	3	9
Yogurt (yogurt drinks)/g	33	23	22	27	25	28

**Table A2:** Gastrointestinal items from Toddler Gut Comfort Questionnaire at baseline, on day 7 and day 14 among PPS subjects.

Toddler Gut Comfort Questionnaire Item	Total	A2 GUM	Conventional Milk	Adjusted Mean Difference (95% CI)	*p*
	(*n* = 308)	(*n* = 180)	(*n* = 128)		
**BASELINE**					
**Total GCS**	15.1 ± 5.3	15.0 ± 5.2	15.2 ± 5.5	/	0.74
**Gastrointestinal Symptoms**					
Stooling issues *	1.5 ± 0.8	1.5 ± 0.8	1.5 ± 0.9	/	0.54
Constipation *	1.5 ± 0.9	1.5 ± 0.9	1.5 ± 0.9	/	0.68
Diarrhea *	1.3 ± 0.6	1.3 ± 0.6	1.3 ± 0.6	/	0.85
Gassiness *	1.4 ± 0.7	1.3 ± 0.6	1.4 ± 0.8	/	0.19
Abdominal pain *	1.2 ± 0.6	1.3 ± 0.6	1.2 ± 0.5	/	0.83
Bloated †	1.3 ± 0.7	1.3 ± 0.7	1.4 ± 0.7	/	0.66
**Gastrointestinal Related Behaviors**					
Fussy and irritable *	1.6 ± 0.9	1.5 ± 0.8	1.6 ± 1.0	/	0.32
Sleep problem ‡	1.5 ± 0.8	1.5 ± 0.7	1.6 ± 0.9	/	0.75
Sleepy during the day *	1.5 ± 0.7	1.5 ± 0.8	1.5 ± 0.7	/	0.88
Waking up during the night §	2.2 ± 1.0	2.2 ± 1.0	2.1 ± 0.9	/	0.39
**DAY 7**					
**Total GCS**	14.9 ± 5.5	14.8 ± 5.0	15.0 ± 6.1	−0.13 (−1.12, 0.86)	0.80
**Gastrointestinal Symptoms**					
Stooling issues *	1.5 ± 0.8	1.4 ± 0.8	1.5 ± 0.8	−0.003 (−0.17, 0.16)	0.97
Constipation *	1.5 ± 0.8	1.4 ± 0.7	1.5 ± 0.9	−0.08 (−0.21, 0.06)	0.27
Diarrhea *	1.3 ± 0.6	1.2 ± 0.5	1.4 ± 0.6	−0.13 (−0.2, −0.02)	**0.027**
Gassiness *	1.3 ± 0.5	1.3 ± 0.6	1.5 ± 0.9	−0.05 (−0.18, 0.07)	0.41
Abdominal pain *	1.3 ± 0.6	1.3 ± 0.5	1.3 ± 0.6	−0.06 (−0.17, 0.05)	0.28
Bloated †	1.3 ± 0.6	1.3 ± 0.5	1.4 ± 0.7	−0.07 (−0.19, 0.05)	0.24
**Gastrointestinal Related Behaviors**					
Fussy and irritable *	1.5 ± 0.8	1.4 ± 0.7	1.5 ± 0.8	−0.03 (−0.16, 0.11)	0.70
Sleep problem ‡	1.5 ± 0.7	1.5 ± 0.7	1.5 ± 0.7	−0.03 (−0.18, 0.12)	0.69
Sleepy during the day *	1.5 ± 0.6	1.5 ± 0.7	1.5 ± 0.6	0.01 (−0.12, 0.14)	0.92
Waking up during the night §	2.2 ± 1.0	2.3 ± 1.0	2.1 ± 0.9	0.19 (0.03, 0.35)	**0.022**
**DAY 14**					
**Total GCS**	14.1 ± 4.8	14.0 ± 4.3	14.3 ± 5.5	−0.25 (−1.13, 0.64)	0.59
**Gastrointestinal Symptoms**					
Stooling issues *	1.4 ± 0.7	1.3 ± 0.6	1.5 ± 0.9	−0.10 (−0.24, 0.04)	0.15
Constipation *	1.4 ± 0.7	1.3 ± 0.6	1.4 ± 0.9	−0.16 (−0.2, −0.04)	**0.010**
Diarrhea *	1.3 ± 0.5	1.2 ± 0.4	1.3 ± 0.5	−0.06 (−0.17, 0.04)	0.22
Gassiness *	1.3 ± 0.6	1.2 ± 0.4	1.3 ± 0.6	−0.08 (−0.19, 0.03)	0.15
Abdominal pain *	1.2 ± 0.5	1.2 ± 0.4	1.3 ± 0.5	−0.07 (−0.17, 0.03)	0.16
Bloated †	1.3 ± 0.5	1.2 ± 0.4	1.3 ± 0.6	−0.11 (−0.22, −0.01)	**0.038**
**Gastrointestinal Related Behaviors**					
Fussy and irritable *	1.4 ± 0.7	1.4 ± 0.7	1.4 ± 0.7	0.01 (−0.12, 0.15)	0.83
Sleep problem ‡	1.4 ± 0.7	1.4 ± 0.7	1.4 ± 0.6	−0.01 (−0.15, 0.13)	0.89
Sleepy during the day *	1.4 ± 0.6	1.4 ± 0.6	1.4 ± 0.6	−0.02 (−0.14, 0.10)	0.77
Waking up during the night §	2.2 ± 1.0	2.3 ± 1.1	1.9 ± 0.9	0.25 (0.07, 0.43)	**0.006**

* 1-Never, 6-Always; † 1-None, 6-Very strong; ‡ 1-Not a problem at all, 6-A very serious problem; § 1-Never, 2-Once, 3-Twice, 4-Three Times, 5-Four times, 6-More than four times. *p* values < 0.05 were marked in bold.
